# Deficiency of prion protein induces impaired autophagic flux in neurons

**DOI:** 10.3389/fnagi.2014.00207

**Published:** 2014-08-25

**Authors:** Hae-Young Shin, Jeong-Ho Park, Richard I. Carp, Eun-Kyoung Choi, Yong-Sun Kim

**Affiliations:** ^1^Ilsong Institute of Life Science, Hallym UniversityAnyang, Gyeonggi-do, South Korea; ^2^New York State Institute for Basic Research in Developmental DisabilitiesStaten Island, NY, USA; ^3^Department of Biomedical Gerontology, Graduate School of Hallym UniversityChuncheon, Gangwon-do, South Korea; ^4^Department of Microbiology, College of Medicine, Hallym UniversityChuncheon, Gangwon-do, South Korea

**Keywords:** prion protein, autophagy, autophagic flux, lipofuscin pigmented autophagy, *Prnp*-deficient mice

## Abstract

Normal cellular prion protein (PrP^C^) is highly expressed in the central nervous system. The Zürich I *Prnp*-deficient mouse strain did not show an abnormal phenotype in initial studies, however, in later studies, deficits in exploratory behavior and short- and long-term memory have been revealed. In the present study, numerous autophagic vacuoles were found in neurons from Zürich I *Prnp*-deficient mice. The autophagic accumulation in the soma of cortical neurons in Zürich I *Prnp*-deficient mice was observed as early as 3 months of age, and in the hippocampal neurons at 6 months of age. Specifically, there is accumulation of electron dense pigments associated with autophagy in the neurons of Zürich I *Prnp*-deficient mice. Furthermore, autophagic accumulations were observed as early as 3 months of age in the CA3 region of hippocampal and cerebral cortical neuropils. The autophagic vacuoles increased with age in the hippocampus of Zürich I *Prnp*-deficient mice at a faster rate and to a greater extent than in normal C57BL/6J mice, whereas the cortex exhibited high levels that were maintained from 3 months old in Zürich I *Prnp*-deficient mice. The pigmented autophagic accumulation is due to the incompletely digested material from autophagic vacuoles. Furthermore, a deficiency in PrP^C^ may disrupt the autophagic flux by inhibiting autophagosome-lysosomal fusion. Overall, our results provide insight into the protective role of PrP^C^ in neurons, which may play a role in normal behavior and other brain functions.

## Introduction

Normal PrP^C^ is an extracellular surface glycosylphosphatidylinositol-anchored glycoprotein that is highly expressed in the central nervous system, including the hippocampus and cortical areas (Prusiner, 1991, 1998). Prions cause transmissible neurodegenerative diseases that are associated with the accumulation of PrP^Sc^, a misfolded and aggregated form of PrP^C^ (Kretzschmar et al., 1986; Stahl et al., 1987; Prusiner, 1991; Pan et al., 1993; Klionsky et al., 2012).

PrP^C^ plays a role in cell metabolism and maintenance including neurotransmitter metabolism, signal transduction, copper (Cu) metabolism, cell adhesion, neuritogenesis, and antioxidant activity (Brown et al., 1999; Herms et al., 1999; Mangé et al., 2002; Krebs et al., 2007; Lobão-Soares et al., 2007). To address the function of PrP^C^, several mouse lines deficient in PrP^C^ (*Prnp-*deficient) have been independently generated; interestingly, these lines exhibited different phenotypes (Büeler et al., 1992; Manson et al., 1994; Moore et al., 1999; Prestori et al., 2008). Specifically, Nagasaki (Ngsk) and Rcm0 *Prnp-*deficient mice develop ataxia in old age, due to cerebellar Purkinje cell degeneration (Sakaguchi et al., 1996; Moore et al., 1999; Prestori et al., 2008), whereas the other lines; i.e., Zürich I and Edinburgh (Edbg) *Prnp-*deficient mice, never exhibit this abnormal phenotype (Büeler et al., 1992; Klamt et al., 2001). The demyelination occurred without clinical symptoms in the peripheral nervous systems of the aging mice (Weissmann and Flechsig, 2003). Moreover, in contrast to Ngsk and Rcm0 *Prnp-*deficient cells, the Zürich I and Edbg lines did not upregulate Doppel, a PrP homologue that induces cerebellar degeneration (Moore et al., 1999; Prestori et al., 2008).

Mouse models in which the PrP^C^ gene is ablated have been used to examine behavior and cognition in several studies. The first studies of Zürich I *Prnp-*deficient mice suggested that these animals have no gross anatomical abnormalities in the brain and visceral organs and that the deficiency of this cellular protein was insufficient to cause significant behavioral abnormalities (Büeler et al., 1992; Manson et al., 1994). However, more recent studies show differences between *Prnp-*deficient and wild-type (WT) mice (Martins et al., 2002; Spudich et al., 2005; Xikota et al., 2008).

*Prnp*-deficient mice exhibit an increased predilection to seizures, motor and cognitive abnormalities, reduced synaptic inhibition and long-term potentiation in the hippocampus. Moreover, these lines exhibit altered development of the granule cell layer, misregulation of the cerebellar network, and age-dependent spongiform changes with reactive astrogliosis (Weissmann and Flechsig, 2003; Criado et al., 2005). Additionally, *Prnp*-deficient hippocampal neurons exhibit increased susceptibility to damage from oxidative stress (Oh et al., 2008). In addition, learning and memory impairments have been demonstrated in the cognitive performance of *Prnp-*deficient mice (Criado et al., 2005; Nazor et al., 2007). Normal hippocampal function is required for spatial recognition memory and hippocampal neurons may play a critical role in the formation of internal representations and the spatial organization of the environment (Barnes, 1979; Eichenbaum et al., 1992).

A certain degree of neurodegeneration is induced by autophagic cell death, which is characterized by the accumulation of autophagic vacuoles, including phagophores, autophagosomes, and autolysosomes (Petersén et al., 2001; Ko et al., 2005; Sanchez-Varo et al., 2012). Although autophagy plays an important role in the normal maintenance of cellular homeostasis (i.e., the turnover of intracellular organelles and long-lived proteins), excessive autophagy is also proposed to cause cellular destruction (Ko et al., 2005; Klionsky et al., 2008, 2012; Odagiri et al., 2012). During this process, the cytoplasmic form of microtubule-associated light chain 3 (LC3-I, 18 kDa) is converted into phagophores and the autophagosomal membrane-bound form of LC3 (LC3-II, 16 kDa), which is the most reliable marker for activation of autophagy (Kabeya et al., 2000; Klionsky et al., 2008, 2012).

In previous *in vitro* studies, we reported that autophagy is upregulated in serum deprived *Prnp-*deficient hippocampal neurons and under hydrogen peroxide-induced oxidative stress (Oh et al., 2008, 2012). To further investigate a mechanism for the protective role of PrP^C^ in an *in vivo* system, we examined the impaired autophagic flux in neurons of Zürich I *Prnp-*deficient mice during aging. In our study, we demonstrated that *Prnp-*deficient mice exhibit altered ultrastructural changes in hippocampal and cerebral cortical neurons including abnormal autophagic vacuoles compared to age-matched WT mice.

## Materials and methods

### Animals

The Zürich I *Prnp*-deficient mice were kindly provided by Dr. A. Aguzzi (Institute of Neuropathology, University Hospital of Zürich, Switzerland). The Zürich I *Prnp*-deficient mice were maintained as inbred strains in the animal facility of the Ilsong Institute of Life Science. Pathogen-free Zürich I *Prnp*-deficient mice and C57BL/6J mice were housed in a clean facility under natural light-dark cycle conditions (12 h each of light and dark). The WT control (C57BL/6J) and experimental (Zürich I) mice were examined at 3 months, 6 months and 12 months of age. Eighteen mice (WT and Zürich I mice age groups: 3 months, *n* = 3; 6 months, *n* = 3; 12 months, *n* = 3) were fixed for electron microscopic analysis, and 36 mice (two separate experiments of WT and Zürich I mice age groups: 3 months, *n* = 3; 6 months, *n* = 3; 12 months, *n* = 3) were used for Western blot analysis. The animal protocol was approved by the Hallym Medical Center Institutional Animal Care and Use Committee (HMC2012-0-1107-3).

### Transmission electron microscopic analysis (TEM analysis)

The animals were sacrificed by perfusion with 4% paraformaldehyde and PBS-buffered 2.5% glutaraldehyde (0.1 M, pH 7.4) under deep anesthesia with 16.5% urethane. The hippocampus and cerebral cortex were trimmed into small pieces immediately following surgical removal and were kept in the same fixative (4% paraformaldehyde and PBS-buffered 2.5% glutaraldehyde) for 2 h on ice. Post-fixation was performed in 1% PBS-buffered osmium tetroxide, followed by dehydration through a graded ethanol and embedding in epoxy resin (Epon 812 kits; EMS, PA, USA). Ultra-thin sections (75 nm) were cut with an RMC MTXL ultramicrotome (Tucson, Arizona, USA) and then stained with uranyl acetate and lead citrate. The sections were examined with a JEM-1011 transmission electron microscope (TEM; JEOL, Tokyo, Japan).

### Western blot analysis

The frozen hippocampus and cerebral cortex of C57BL/6J and Zürich I *Prnp*-deficient mice were gently homogenized using a glass-homogenizer (Corning, New York, USA) in 10 vol. of homogenizing buffer [50 mM Tris-HCl pH 7.4, 150 mM NaCl, 1 mM EDTA, 0.25% Na-deoxycholate, 1% NP-40, protease inhibitor cocktail (Roche, Mannheim, Germany)]. The homogenates were rocked at 4°C for 60 min and centrifuged at 5,000 g for 10 min at 4°C to remove cell debris. The supernatant was collected and protein concentration was determined with a BCA protein assay kit (Pierce, Rockford, IL, USA). Equal amounts of protein were boiled in 2X SDS gel-loading buffer for 5 min and then separated by SDS-PAGE using 12% or 15% acrylamide gels. The proteins separated by gel electrophoresis were transferred to nitrocellulose membranes (Amersham Pharmacia Biotech, Piscataway NJ, USA) using an electrophoretic transfer system (Bio-Rad, California, USA) at 60 V for 2 h. The membranes were washed with Tris-buffered saline solution (TBST, pH 7.6 0.05% Tween 20), then blocked for 1 h in TBS containing 5% skim milk. The membranes were then incubated overnight at 4°C with primary antibodies against LC3B (1:2,000; Sigma-Aldrich, St. Louis, Missouri, USA) and β-Actin (1:10,000). After washing, the membranes were incubated with horseradish peroxidase (HRP)-conjugated secondary antibodies against rabbit and mouse IgG antibodies (1:5,000; Thermo Scientific, Rockford, IL, USA) for 40 min at room temperature. The membranes were then washed again, and bound secondary antibodies were visualized using enhanced chemiluminescence (Thermo Scientific, Rockford, IL, USA) according to the manufacturer’s instructions. The band intensity was determined by densitometric scanning using Bio-Rad Quantity One software (Bio-Rad, Hercules, California, USA).

### Immunogold electron microscopy (immuno-EM)

Nickel grids with epoxy-embedded ultra-thin sections (75 nm) were used for antigen retrieval. The sections were immersed in target retrieval solution (TRS; Dako, Glostrup, Denmark) and incubated for 15 min at 110°C, then incubated in blocking solution for 20 min. The sections were then incubated with rabbit polyclonal anti-LC3B antibody (Sigma-Aldrich, St. Louis, Missouri, USA; 1:100 in 0.5% BSA, 0.5 M NaCl, with PBS) for 2 h at 55°C. After washing (0.5% BSA, 0.5 M NaCl, 0.1% gelatin, 0.05% Tween-20 in PBS), the sections were incubated for 2 h at 50°C with goat anti-rabbit IgG-conjugated 10 nm gold particles (Aurion, Wageningen, NL; diluted 1:30 in 0.5% BSA, 0.5 M NaCl, with PBS). After washing (0.5% BSA, 0.5 M NaCl, 0.1% gelatin, 0.05% Tween-20 in PBS), all of the sections were counterstained with uranyl acetate in the usual manner. The sections were examined with a JEM-1011 TEM (JEOL, Tokyo, Japan).

## Results

### Autophagic vacuoles increased in hippocampus and cerebral cortex of young Zürich I *Prnp*-deficient mice

**Figure 1:**
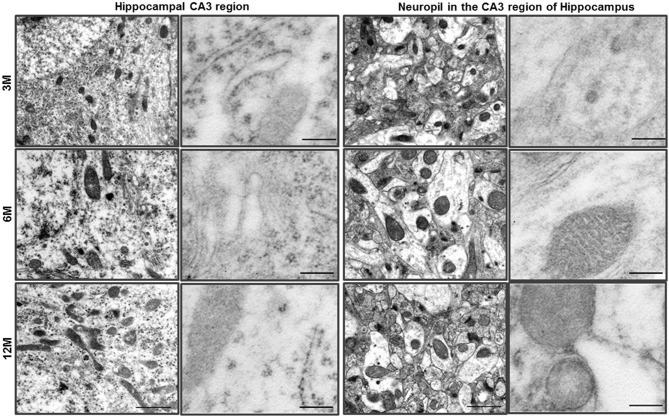
**Ultrastructural analysis of the hippocampus in C57BL/6J mice**. The neurons of the hippocampal CA3 region of C57BL/6J mice exhibited a pale nucleus and normal organelles. LC3B immunogold particles were not observed in the cytosol. The neuropil in the CA3 region of hippocampus exhibited normal neurites and mitochondria, and LC3B immunogold particles were not observed. Scale bars, 1 μm for typical TEM; 200 nm for immuno-EM.

**Figure 2:**
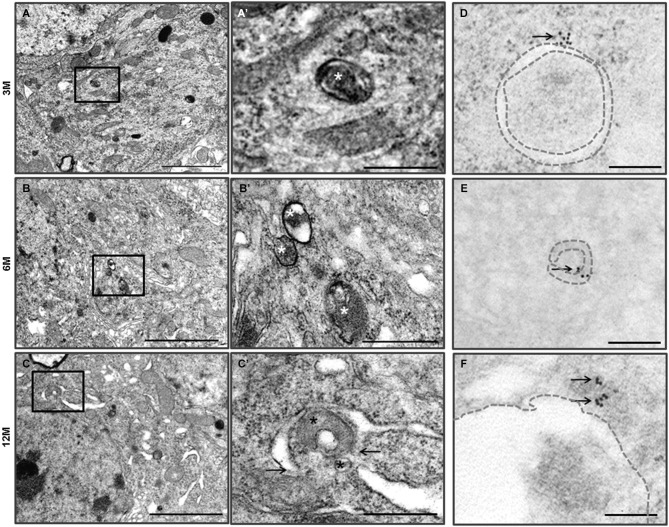
**Autophagy in the hippocampal neurons of the Zürich I** ***Prnp*-deficient mice. (A–C)** The vacuoles (asterisks) were bounded by single and double (arrows) membranes and contained amorphic cytoplasmic residues. The images in **(A’–C’)** were taken at a higher magnification compared to the solid line boxes in **(A–C)**. **(D–F)** LC3B immunogold particles (arrows) were localized on the membrane (dotted lines) and lumens of vacuoles in the degenerated hippocampal neurons, which were undergoing autophagy. Scale bars, 2 μm for **(A–C)**; 500 nm for **(A’–C’)**; 200 nm for **(D–F)**.

We previously reported that *Prnp*-deficient hippocampal neuron cells were more susceptible to autophagic induction than WT control cells under external environmental changes such as serum deprivation and oxidative stress (Oh et al., 2008, 2012). Furthermore, we used TEM to assess the morphology of the hippocampus from the two mouse lines. WT control mice (C57BL/6J) exhibited a pale nucleus with normal mitochondria, endoplasmic reticulum (ER) and Golgi apparatus in three age groups (Figure 1). Occasionally, the hippocampal neurons of 12-month-old C57BL/6J mice exhibited a small number of vacuoles. Zürich I hippocampal neurons exhibited a normal appearance only in 3-month-old mice, in which a small number of vacuoles were observed, similar to the 12-month-old C57BL/6J hippocampus. However, in 6- and 12-month-old Zürich I mice, the hippocampal neurons had a partially electron-dense nucleus and cytoplasm. Moreover, these cells contained normal mitochondria and Golgi and partially swelled ER with vacuoles of various sizes (Figure 2). The vacuoles in Zürich I *Prnp*-deficient hippocampal neurons were surrounded by single and double membranes and contained degraded mitochondria and amorphic cytoplasmic residues (Figures 2A’–C’). The structure of these vacuoles was characteristic of autophagy. We then analyzed the autophagic vacuoles using immuno-EM with an anti-LC3B antibody. LC3B reactive immunogold particles were localized with the single and double membranes (Figures 2D–F).

**Figure 3:**
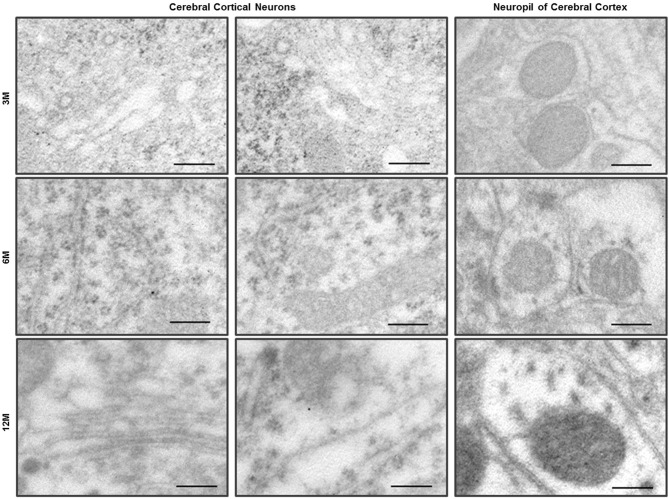
**Immuno-EM analysis with anti-LC3B antibody in the cerebral cortex of C57BL/6J mice**. LC3 immunogold particles were not observed on the neurons and neurites in the neuropil of cerebral cortex. Scale bars, 200 nm.

**Figure 4:**
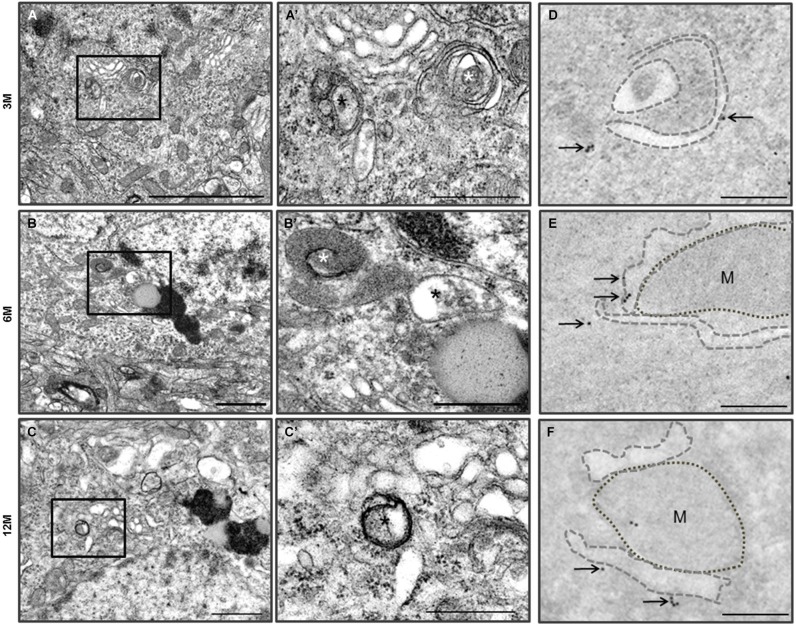
**Autophagy in the cerebral cortical neurons of the Zürich I** ***Prnp*-deficient mouse strains. (A–C)** The vacuoles (asterisks) were bounded by single electron dense membranes and double and multilayered membranes. They contained amorphic cytoplasmic residues and degraded small organelles. The images in **(A’–C’)** were taken at a higher magnification compared to the solid line boxes in the **(A–C)**. **(D–F)** LC3B immunogold particles (arrows) were localized on the membranes and phagophores (dotted lines) in the degenerated cortical neurons, which were undergoing autophagy. M: mitochondria. Scale bars, 2 μm for **(A)**; 1 μm for **(B–C)**; 500 nm for **(A’–C’)**; 200 nm for **(D–F)**.

Ultrastructural observation of cerebral cortexes from the C57BL/6J and Zürich I *Prnp*-deficient mouse lines was carried out. WT control mice exhibited healthy neurons with normal mitochondria, ER and Golgi apparatus at the three age groups and LC3B immunogold particles were rarely detected only in the cytosol of neurons and neurites by immune-EM analysis (Figure 3). Interestingly, many vacuoles were observed in cortical neurons of Zürich I mice from 3 months of age (Figures 4A,A’). Vacuoles are an amorphic matrix surrounded by single and double membranes (Figures 4B,B’,C,C’). These vacuoles are associated with LC3B immunogold particles, which means these vacuoles are autophagic vacuoles (Figures 4D–F). These results indicated that a lack of PrP induced the accumulation of autophagosomes existed as early as 3 months old in cortical neurons and at 6 months old in hippocampal neurons.

### Accumulation of autophagy associated with lipofuscin pigments in hippocampal and cerebral cortical neurons of Zürich I *Prnp*-deficient mice

**Figure 5:**
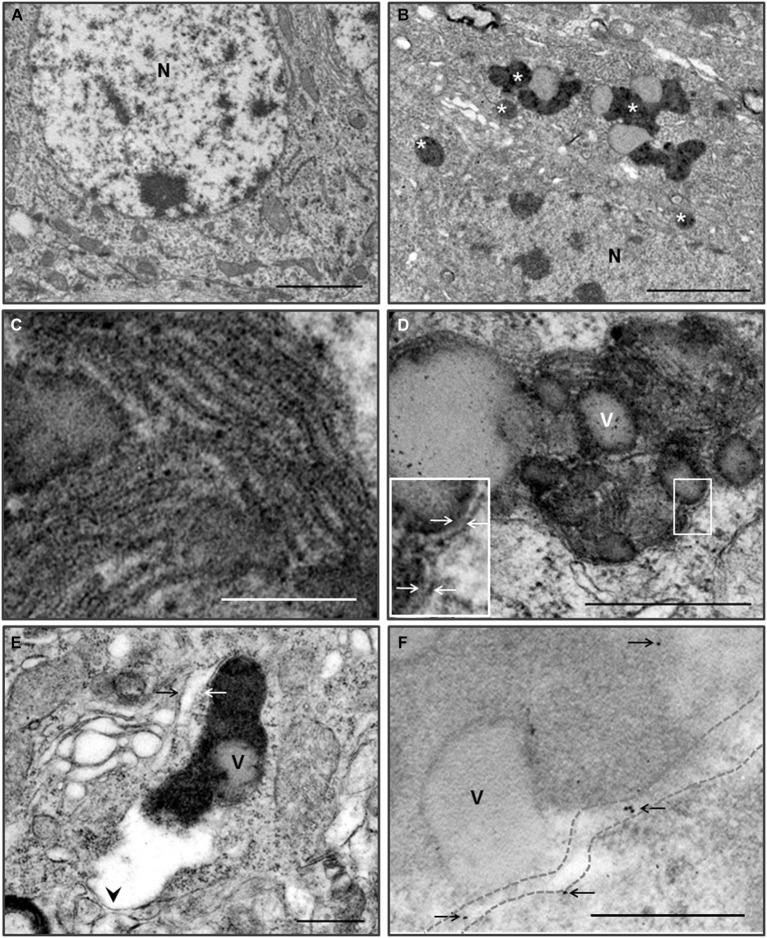
**Pigmented autophagy in the hippocampal and cortical neurons of the Zürich I** ***Prnp*-deficient mice**. **(A)** Low magnification of hippocampal neuron of WT mice at 6 month-old. **(B)** Low magnification of hippocampal neuron of Zürich I mice at 6 month-old. The neurons contain numerous electron dense pigments and electron-lucent vacuole complex structures (asterisks). N, nucleus. **(C)** Higher magnification of the lamellar structures of the lipofuscin pigmented substance, which were pentalaminar structures. **(D)** The neurons exhibited lipofuscin pigment and electron-lucent vacuoles (v), which were bounded by double membranes (arrows on the higher magnification of solid line boxes). **(E)** The fine granular pigmented autophagic areas were bounded by single (arrowhead) and double (arrows) membranes. **(F)** LC3B gold particles (arrows) were localized on the membrane (dotted lines) and in the pigmented electron dense matrix in the cortical neurons of Zürich I *Prnp*-deficient mice. Scale bars, 2 μm for **(A,B)**; 100 nm for **(C)**; 500 nm for **(D–F)**.

Many neurons in young Zürich I *Prnp*-deficient mice contain electron dense pigments and electron-lucent vacuole complex structures (Figures 5A,B). Most of the pigments have been associated with one or more vacuoles. The features of pigmented structures indicate that lipofuscin granules are involved. The lipofuscin granules were round or irregular in shape, and the inner structure of the dense matrix was heterogeneous. The most common inner structure was a fine granular substance, as well as a coarse granular substance consisting of fine granules and a lamellar structure (Figures 5D,E). The lamellar structures were a repeated pattern of five bands (pentalaminar structure) that made up the dense lamella in the center, the electronlucent lamellar band next to the central lamella and the dense lamella outer band (Nunomura and Miyagishi, 1993; Figure 5C).

The lipofuscin granules were surrounded by single and double membranes (Figures 5D,E). The pigmented vacuoles were reminiscent of autophagy. We analyzed the pigmented vacuoles using immuno-EM with an anti-LC3B antibody. LC3B immunogold particles were localized on the pigmented vacuolar membrane and the electron dense matrix (Figure 5F). These results indicated that the pigmented vacuoles are autophagic vacuoles, and suggest a deficiency in PrP-induced pigmented autophagic accumulation in the hippocampus and cerebral cortex of young Zürich I *Prnp*-deficient mice.

### Early autophagic neuronal processes were observed in neuropil of the CA3 region of hippocampus and the cerebral cortex of Zürich I *Prnp*-deficient mice

**Figure 6:**
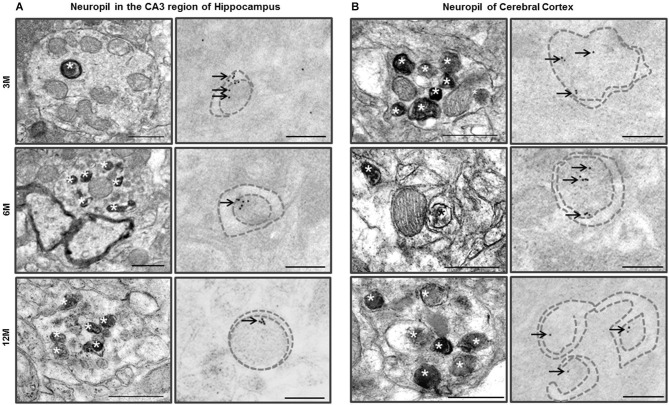
**Ultrastructural observation of neuropils in the CA3 region of hippocampus and cerebral cortex of Zürich I mice**. **(A)** The neurites in neuropil of the CA3 region of hippocampus contained many electron dense inclusion bodies (asterisks); these features are indicative of high autophagy activity of neurites. Additionally, LC3B immunogold particles (arrows) localized on the autophagic membrane (dotted lines). **(B)** The neuritis contained double membrane bound vacuoles and many electron dense inclusion bodies (asterisks) as early as at 3 months old in cortical neurites. LC3B immunogold particles (arrows) localized on the membrane (dotted lines) and lumen of autophagic vacuoles. Scale bars, 500 nm for typical TEM; 200 nm for immuno-EM.

TEM analysis of the neuropil in the CA3 region of hippocampus of the Zürich I *Prnp*-deficient mouse strain revealed that the neurites contained single, double or multilayered vacuoles. Interestingly, we also found that subsets of the unmyelinated and myelinated neurites in the neuropil of the CA3 region of hippocampus and cerebral cortex exhibited double membrane vacuoles and many electron dense inclusion bodies in the Zürich I mice (Figure 6). The hippocampal unmyelinated neurite subsets occasionally contained a few electron dense inclusion bodies (Figure 6A), whereas cortical neurite subsets exhibited many electron dense inclusion bodies in Zürich I mice at 3 months old (Figure 6B). These morphological features, such as electron dense inclusion bodies in neurite subsets, are characteristic of the high autophagic activity (Heitz et al., 2010; Sanchez-Varo et al., 2012).

We assessed whether LC3 is associated with the vacuoles of hippocampal and cortical neurites. The LC3B immunogold particles were rarely detected in the neurites of C57BL/6J neuropil in the CA3 region of hippocampus and cerebral cortex. In contrast, in Zürich I *Prnp*-deficient mice, the LC3B immunogold particles were associated with the membranes and lumens of neuritic vacuoles in three age groups (Figure 6). These results suggest that PrP deficiency induced autophagic activity of neurites in young mice as early as 3 months of age.

### Expression of LC3-II increased in the brains of Zürich I *Prnp*-deficient mice

**Figure 7:**
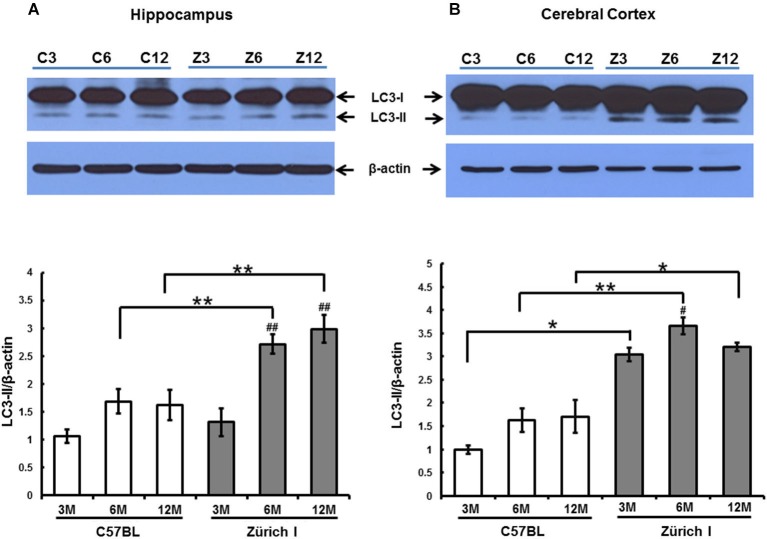
**The expression level of LC3-II in 3-month-old, 6-month-old and 12-month-old C57BL/6J and Zürich I mouse strains as determined using Western blot analysis**. **(A)** The LC3 protein was identified as a double band (i.e., LC3-I and LC3-II). The level of LC3-II in the hippocampus of Zürich I mice was significantly increased at 6 months and 12 months of age compared to 3 months (^##^ *p* < 0.001) and was significantly higher than for C57BL/6J mice at both 6 months and 12 months of age (** *p* < 0.001). **(B)** The level of LC3-II in the cerebral cortex of Zürich I mice was significantly higher than for C57BL/6J mice (* *p* < 0.01, ** *p* < 0.001). The level of LC3-II in the cortex of Zürich I mice was significantly increased at 6 months of age compared to 3 months of age (^#^ *p* < 0.01). Thirty-six mice (2 separate experiments of WT and Zürich I mice age groups: 3 months, *n* = 3; 6 months, *n* = 3; 12 months, *n* = 3) were used. These results are representative of at least two separate experiments.

Accordingly, we analyzed whether autophagy is also induced by a lack of PrP in the hippocampus of Zürich I mice using the anti-LC3B antibody using Western blot analysis. Two LC3 family members (LC3A and LC3B) undergo a characteristic C-terminal cleavage and are binds tightly to the autophagic membranes (Wu et al., 2006). The N-terminal domain of LC3 is an essential component of autophagy that acts as an adaptor protein between microtubules and autophagosomes (Kouno et al., 2005). In under elevated levels of autophagic vacuoles condition, LC3B levels increased, but not LC3A (Klionsky et al., 2008). This supports that LC3A and LC3B display different functions. We therefore used LC3B antibody as an autophagosomal marker. During aging of the hippocampus, the expression levels of the LC3B-I were unchanged in all of the mouse strains at all ages (Figure 7A). In the hippocampus of C57BL/6J mice, LC3B-II expression levels did not change with age either. However, the amount of LC3B-II in Zürich I mice significantly increased at 6- and 12-months compared to 3-month-old mice; these levels were significantly higher than C57BL/6J mice at 6- and 12-months of age (Figure 7A).

In the cerebral cortex, the expression levels of LC3B-I were unchanged in all experimental animals and were similar to the levels of LC3B-I in the hippocampus. The pattern of LC3B-II expression levels in the cortex differed from the pattern in the hippocampus in Zürich I mice. LC3-II exhibited a high level of expression that was maintained during aging from 3 month old in Zürich I mice (Figure 7B).

These results suggest that a PrP deficiency induced high expression levels of LC3-II in the hippocampus and cerebral cortex in young Zürich I mice, at 3 months of age.

## Discussion

Here, we report an accumulation of autophagosomes in neuronal processes as early as at 3 months of age in both the hippocampus and cerebral cortex of Zürich I *Prnp*-deficient mice. TEM and immuno-EM analyses revealed autophagic compartments within neuronal processes in neuropil of the CA3 region of hippocampus and cerebral cortex of Zürich I mice at 3 months old. High autophagic activity of neurites has also been recognized as an early event in many neurodegenerative diseases, including prion disease (Ferrer, 2002).

PrP^C^ has been noted as an important factor for normal hippocampal and cortical synaptic function (Collinge et al., 1994; Mouillet-Richard et al., 2000; Fuhrmann et al., 2007). PrP^C^ was observed predominantly in the neuropil, with particular localization to the synaptic membranes in all hippocampal layers (Herms et al., 1999; Fournier et al., 2000). In murine prion disease, the appearance of the earliest behavioral deficit is correlated to a loss of synapses in the hippocampus, prior to detectable neuronal loss (Cunningham et al., 2003). Early autophagic accumulation of neurites was observed in neuropil in the CA3 region of hippocampus and cerebral cortex in 3-month-old Zürich I *Prnp*-deficient mice in the present analyses. These ultrastructural results are similar to those observed for cerebellar Purkinje cells of the Ngsk *Prnp*-deficient mice, where autophagy accumulated in the neuronal processes occurs prior to significant Purkinje cell loss at 3 months of age (Heitz et al., 2010). The autophagy that was observed in the neurites of Zürich I *Prnp*-deficient hippocampal CA3 region and cortical neuropils could be viewed as an initial step in autophagy-induced cell degeneration or as a protective reaction of neurons to maintain neurite homeostasis (Komatsu et al., 2007). Physiological alterations within *Prnp*-negative neurites could locally trigger autophagic machinery.

Interestingly, we observed that the membrane-surrounded electron dense pigment complex induced by lipofuscin, with the neurons of Zürich I *Prnp*-deficient mice. Lipofuscin shared common features, particularly in their electron dense components and electron-lucent components. Particularly, the lamellar structure of the electron dense granular substance demonstrated that pentalaminar structures were exactly common to the lipofuscin granules (Nunomura and Miyagishi, 1993). Lipofuscin granule morphology is divided into four types described using the cerebral cortex neuron of senile and vitamin E-deficient rats (Miyagishi et al., 1967). Type 1, shows a fine granular and electron dense component with or without vacuoles. Type 2, is homogeneous and structureless and is surrounded by an indistinct membrane without vacuoles. Type 3, is quite low and has a lamellar structure similar to mitochondrial cristae, which shows very few vacuoles. Type 4, is compound where the three types are found in various combinations (Miyagishi et al., 1967). In general, accumulation occurs over a long life producing pigmented autophagic vacuoles in contrast with the short life of typical autophagic vacuoles (Sulzer et al., 2008). However, in our study, pigmented autophagy was observed in young Zürich I *Prnp*-deficient mice.

There are two morphological causes related to the pigmented autophagic accumulation in young *Prnp*-deficient mice: an accumulation of lipofuscin early in age and an accumulation of incomplete digestible materials. Lipofuscin, which is known as an aging pigment, accumulates in neurons and glia during aging (Goyal, 1982), is highly insoluble and reactive, consists of proteins and lipids, and is highly enriched in iron and other metals (Jolly et al., 2002; Sulzer et al., 2008). Metal uptake, transport, and dyshomeostasis induced lipofuscin granules caused by the lack of PrP^C^ and, finally, pigmented autophagic accumulation in young mice. Cu is essential for normal development of the nervous system and plays a crucial role in myelin production and maintenance (Gybina et al., 2009). Cu deficiency impairs brain development caused by mitochondrial dysfunction (Mercer, 1998). The WT neurons showed resistance to Cu toxicity in comparison with neurons lacking PrP^C^ (Vassallo and Herms, 2003). Nevertheless, Cu and zinc concentrations were not significantly altered; however, iron was significantly reduced in *Prnp*-deficient brains compared with WT brains (Pushie et al., 2011). The altered iron-uptake in the gut and iron deficiency in indicates that PrP^C^ maintains metal uptake and transport (Singh et al., 2009, 2013). Furthermore, PrP^C^ is a ferrireductase and maintains a role in the homeostasis of cellular iron (Singh et al., 2013). Therefore, the aggregations of PrP^C^ and iron dyshomeostasis are causally related in sporadic Creutzfeldt-Jakob disease, Parkinson’s disease and Alzheimer’s disease (Bandyopadhyay et al., 2013; Singh et al., 2014). The altered metal levels seen in *Prnp*-deficient mice demonstrate that neuronal function was impaired although there was a relatively mild phenotype variation (Tobler et al., 1996).

The cytosolic proteins and organelles are sequestered by a double membrane vacuole and then fuse with lysosomes; these pathways are referred to as “autophagic flux”. This flux is dependent on lysosomal function (Settembre et al., 2013). Aggregated abnormal PrP^C^ stimulates ER stress and affects degradation of MAP2, which inhibits microtubule assembly (Li et al., 2011; Zhang and Dong, 2012). Consequently, disruption of microtubule structure in *Prnp*-deficient mice is related to the final phenomenon of inhibition of lysosomal fusion with autophagosomes. It has been suggested that incompletely digested intracellular organelles become pigmented autophagy in neurons during impaired autophagic flux because of the inhibition of autophagosome-lysosomal fusion in the neurons of *Prnp*-deficient mice (Brunk et al., 1992; Terman and Brunk, 1998; Moreira et al., 2007).

We observed electron dense inclusion bodies in the neuronal processes of Zürich I *Prnp*-deficient mice and these inclusion bodies were the only electron dense materials without electron-lucent vacuoles. Because the lipofuscin pigmented autophagic vacuoles contain lysosomal constituents in the soma of neurons, small electron dense (pigmented) autophagy in the neuronal processes do not have local mature lysosome resources for fusion with autophagic vacuoles (Sulzer et al., 2008). These small, pigmented autophagic vacuoles inhibit neuritic transport and normal secretory operations (Sulzer et al., 2008).

Our results demonstrate that autophagosomes accumulate within the hippocampus and cerebral cortex of young Zürich I *Prnp*-deficient mice. Autophagy is normally induced to maintain cellular homeostasis but can cause cell death when dysregulated (Zhou et al., 2011). The dysregulation of autophagy can adversely affect the turnover of aggregate-prone proteins and defective organelles, potentially contributing to memory impairment in aged mice (Soontornniyomkij et al., 2012). Lack of PrP^C^ in hippocampal CA1 neurons affects the Ca^2+^-activated potassium channel-mediated reduction in Ca^2+^ influx. Importantly, Ca^2+^ influx is known to control several basal cellular functions in neurons and other cell types (Fuhrmann et al., 2006). Therefore, depletion of PrP^C^ causes alterations in the ER (Kyuhou et al., 2006), which forms autophagic vacuoles (Dunn, 1990). We previously reported that under conditions of serum deprivation, the *Prnp*-deficient neuronal cells exhibited increased expression levels of LC3-II and increased accumulation of autophagosomes compared to cells of WT mice (Oh et al., 2008). We further showed that a deficiency of PrP^C^ contributes to hydrogen peroxide (oxidative stress)-induced autophagic cell death in hippocampal neurons via impaired autophagic flux (Oh et al., 2012). Moreover, autophagy induction enhances the elimination of misfolded PrP^C^ prior to accumulation into plaques (Cortes et al., 2012). Taken together, our results suggest that the activation of autophagy in the brain is a pathology-correlated phenomenon in Zürich I *Prnp* -deficient mice.

*Prnp*-deficient mice develop normally, and these animals have no gross anatomical abnormalities in the brain (Büeler et al., 1992), however, extensive studies have revealed numerous subtle alterations in behavior. Expression levels of PrP^C^ in the brain can affect exploratory behaviors, anxiety, locomotor performance and equilibrium, as well as the time required to adapt to novel environments (Lobão-Soares et al., 2007). *Prnp*-deficient mice were also observed to exhibit cognitive deficits and significantly reduced learning and memory compared to controls (Coitinho et al., 2003; Curtis et al., 2003). This is consistent with our TEM results that show high LC3-II expression levels in neurons and neurites and the accumulation of autophagic vacuoles in the hippocampus and cerebral cortex of the Zürich I *Prnp*-deficient mice.

In conclusion, our results revealed that the accumulation of autophagic vacuoles increased in the hippocampus and cortex of Zürich I *Prnp*-deficient mice. The pigmented autophagic accumulation was induced by the increase in lipofuscin granules under PrP-deficient conditions, which is related to incomplete digestion of the molecules of autophagy. Furthermore, the resulting PrP^C^ deficiency interrupts the autophagic flux and consequently the inhibition of normal autophagosome-lysosomal fusion. Overall, our results provide insight into PrP^C^ playing a protective role in neurons and functioning to maintain normal behaviors.

## Conflict of interest statement

The authors declare that the research was conducted in the absence of any commercial or financial relationships that could be construed as a potential conflict of interest.
